# Supplementary material for: Cost-effectiveness evaluation of routine histoplasmosis screening among people living with advanced HIV disease in Latin America and the Caribbean
Source: PLOS Glob Public Health. 2023 Aug 15;3(8):e0001861. doi: 10.1371/journal.pgph.0001861 (PMC10427011; doi:10.1371/journal.pgph.0001861)
Supplement: S4 Table — (DOCX) [file pgph.0001861.s004.docx]

**Supplemental Table 4.** Sensitivity analysis results evaluating cost-effectiveness of *Histoplasma* antigen testing cost of $60 per test.

|  | Cost  (USD) | Incremental Cost | Effectiveness  (life years) | Incremental effectiveness | ICER  (Cost/LYS) |
| --- | --- | --- | --- | --- | --- |
| No Histoplasma antigen screening | $33,763,183 | -- | 423,567 | -- | -- |
| Histoplasma antigen screening | $48,340,176 | $14,576,993 | 507,886 | 84,319 | $173 |
