# Supplementary material for: Cost-effectiveness evaluation of routine histoplasmosis screening among people living with advanced HIV disease in Latin America and the Caribbean
Source: PLOS Glob Public Health. 2023 Aug 15;3(8):e0001861. doi: 10.1371/journal.pgph.0001861 (PMC10427011; doi:10.1371/journal.pgph.0001861)
Supplement: S9 Table — (DOCX) [file pgph.0001861.s009.docx]

**Supplemental Table 9.** Sensitivity analysis results evaluating cost-effectiveness of *Histoplasma* antigen if 30-day mortality for symptomatic histoplasmosis is 25% (treated).

|  | Cost  (USD) | Incremental Cost | Effectiveness  (life years) | Incremental effectiveness | ICER  (Cost/LYS) |
| --- | --- | --- | --- | --- | --- |
| No Histoplasma antigen screening | $33,060,585 | -- | 389,213 | -- | -- |
| Histoplasma antigen screening | $35,059,420 | $1,998,835 | 454,959 | 65,746 | $30 |
