# Supplementary material for: Cost-effectiveness evaluation of routine histoplasmosis screening among people living with advanced HIV disease in Latin America and the Caribbean
Source: PLOS Glob Public Health. 2023 Aug 15;3(8):e0001861. doi: 10.1371/journal.pgph.0001861 (PMC10427011; doi:10.1371/journal.pgph.0001861)
Supplement: Attachment — Submitted filename: LetterOfResponse31March2023.docx [file pgph.0001861.s012.docx]

February 27, 2023

Dear Dr. Kohler,

Thank you for your timely review of our manuscript entitled “Cost-effectiveness evaluation of routine histoplasmosis screening among people living with advanced HIV disease in Latin America and the Caribbean” PGPH-D-23-00160. We very much appreciate the reviewer’s comments and we believe the suggested edits have improved the quality of the manuscript. See below for our point-by-point response.

Reviewer 1:

Comment: Line 138. In order to better understand the paper, it is important to specify the year of the estimate of 256,585 people with HIV in Latin America.

*Response: This figure is taken from the Rajasingham et al paper estimating the Burden of Cryptococcal infection globally (and appropriately cited). While the exact figure is not published in the manuscript, these data are available upon request from the corresponding author. However, we have noted that this estimate pertains to Latin America only. Including the Caribbean, a total of 293,426 have advanced HIV disease. We have updated our model accordingly.*

Comment: Lines 155- 157, 183-187
Check the consistency between points 155 to 157 and 183 to 187. Also, record the parameters to consider a reduction of 85 to 65% of mortality

*Response: Thank you. We have reviewed these lines. Lines155-157 estimate 10% 30-day mortality for persons with asymptomatic histoplasmosis (with or without fluconazole). There are no data in this area, and we highlight the need for further research in the Discussion. Lines 183-184 highlight 13.7% 30-day mortality among symptomatic persons with histoplasmosis who are treated with antifungal therapy. These two estimates are consistent in our opinion. We presumed 65% 30-day mortality for untreated persons with symptomatic histoplasmosis. As stated in the manuscript, the original 85% estimate of 30-day mortality was from a 1963 reference. With the tools of early diagnosis and antiretroviral therapy, our group consensus among histoplasmosis experts was a reduced mortality to 65%. We have added the term ‘expert opinion’ in parenthesis around this estimate.*

Reviewer 2: Rajasingham et al present an interesting analysis that uses simulation modeling to project the cost-effectiveness of using histoplasmosis antigen screening among people with advanced HIV disease (AHD) in Latin America and the Caribbean. This is an important question that is well-suited to be addressed by simulation modeling. The authors have determined some important data sources for several parameters. However, there are a number of assumptions made throughout the parameterization of the decision tree (see Table 1).

Comment: In particular, the assumption that false positives will not be influential needs additional discussion and testing in the model; especially given that data are not provided about the test characteristics of urinary histo antigen for asymptomatic people.

Comment: Assumptions are also made about 100% of people taking the test and acting upon the results, which is not realistic and will overestimate the benefits of a screening program.

Additionally, data have been used from histoplasmosis infection among people without HIV without further discussion regarding whether outcomes would be the same among PWH. To address many of these concerns, the authors could perform one-way sensitivity analyses on parameters to examine the impact of a range of estimates for these assumptions. This is critical to include as per ISPOR-SMDM recommendations. Assessment of multi-ways sensitivity analyses for parameters influential in one-way sensitivity analysis would also be important to include.

*Response: We assumed 100% were screened and treated to really understand the theoretical maximal benefit of such a screening program. We understand that no screening program has 100% efficacy. Because of the reviewer’s comment, we have changed the main output of our model to reflect 80% of eligible persons screened and treated. This is still an optimal scenario but a bit more reflective of the real world.*

*Regarding data from histoplasmosis without HIV infection, we used the best available data. Clearly, there are a lack of data in screening for histoplasmosis in persons with advanced HIV disease. We felt that histoplasmosis outcomes, even among persons without HIV, is more informative than expert opinion alone.*

*Regarding sensitivity analyses, there are an innumerable amount of sensitivity analyses that could be performed. In our experience, the most useful sensitivity analyses are those where the prices are variable, or the parameters are highly variable and would greatly affect the outcome. This is why we chose to evaluate liposomal amphotericin in a sensitivity analysis –this is becoming increasingly available, and the price is so different, it is a policy issue of great importance to stakeholders. There are other variable parameters in our model. For example, the proportion that would be Histoplasma antigen positive and symptomatic, and the outcomes of those asymptomatic Histoplasma antigen positive with and without treatment. However these three parameters have no data to support the estimates. Thus, a sensitivity analysis varying those parameters by an arbitrary percentage is not informative or useful to policymakers.*

*Our final analysis now assumes 80% are screened. A one-way sensitivity analysis explores 100% screened for histoplasmosis to understand the maximum possible effect of a screening program. An additional sensitivity analysis assumes 80% are screened and treated with liposomal amphotericin.*

Additionally, it is not clear that a decision tree is the best modeling approach to use. Because people with AHD are at high risk for a number of opportunistic infections and have extremely high mortality, it is a major assumption that treating people for histoplasmosis will result in long-term survival, as immunosuppressed people with HIV (CD4<200/uL) may will experience a high risk of death from TB, serious bacterial infection, or other diseases. Competing risks of deaths from other highly prevalent comorbidities are not captured in the current approach and are likely to reduce the benefits of screening for one of these comorbidities (i.e., histoplasmosis).

*Response: This is a great point brought up by the reviewer. We have now incorporated competing risks of death within the model. We identified a recent paper from Brazil highlighting the risk of death among people with HIV within 90 days. We identified the initial 3-month risk of death with HIV, and incorporated this into our model. This includes risk of death from other opportunistic infections (most commonly cryptococcal infection and tuberculosis). The risk of other causes of death (such as traffic accidents etc.. is incorporated into the years of life lost calculation –taken from the World Health Organization’s estimates of life expectancy).*

The authors also make the assumption that people with AHD who are treated for histoplasmosis will live for the average life expectancy of people in Brazil; however, people living with HIV in Brazil are at increased risk of experiencing disadvantageous social determinants of health and therefore may be less likely to live to the national average life expectancy.

*Response: Once people with advanced HIV disease have started ART and reconstituted their immune system, their survival is similar to that of a persons without HIV. There may be social determinants of health that may affect clinical outcomes in various countries –though we are modeling Latin America and the Caribbean, which is quite diverse. Thus, arbitrarily lowering the life expectancy estimates may create new inaccuracies. Instead, we have opted to continue to use the WHO life expectancy estimates for Guatemala and Brazil. However, we have added the following statement in the limitations to acknowledge the reviewer’s point:*

*We assumed that those who survived the initial histoplasmosis infection would have the same survival of a person on ART as a person without HIV infection. In reality, social determinants of health may negatively affect life expectancy of PWH.*

The decision tree model does not account for lifetime morbidity/mortality risks among PWH, costs of ART, loss to follow-up from ART and HIV care. Therefore, the extremely attractive estimate of $26/YLS for Histoplasmosis screening in this high-risk population does not include lifetime costs of HIV care or the potential for a shorter life expectancy.

*Response: Correct. This model does not include costs of HIV care, including ART. As the reviewer likely knows, the way ICERs are calculated is to divide the incremental costs by the incremental benefits. Thus, costs such as ART that are included in all strategies will be canceled out. Only the intervention (ie. Histoplasmosis screening and treatment) that is included in one arm, but not the other will be calculated. If we included the costs of HIV care and ART in both arms, they ultimately will be subtracted out, and the result would still be $26/LYS.*

The results are presented as a population finding, as opposed to a per person costs and outcomes, which is not the traditional approach. Importantly, the total costs are presented as the total costs of the program, but do not include any of the other HIV care costs that would be essential if people with HIV survive after screening/treatment for histoplasmosis. Inclusion of these important costs will reduce the cost-effectiveness of the intervention because people who survive will cost more per year than is currently included.

*Response: See above as to why we did not incorporate HIV care costs. Furthermore, it is important to take the perspective of a stakeholder considering investing in a histoplasmosis program. For example, if PEPFAR were to consider histoplasmosis screening and preemptive treatment, our approach would be the best analysis to inform their decision. That investor would not be funding ART, HIV care costs, healthcare workers costs for routine HIV care. That investor would be funding histoplasmosis screening and preemptive treatment.*

*We have designed this model based off a prior model used to evaluate the benefit of cryptococcal screening and preemptive treatment. Our group has had tremendous success advocating for national cryptococcal screening and treatment programs, and we have success in using these models to engage stakeholders and investors.*

A small query is that most modeling analyses that use published data are still reviewed by IRB to confirm that they meet "exempt" criteria.

*Response: This research does not require IRB approval, as it does not involve human subjects. Exempt status applies where there is no more than minimal risk to subjects.*

I am concerned that the analysis as current presented does not accurately portray the full costs or examine the potential cost-effectiveness of such a screening program.

*Response: I hope we have addressed the concerns of this review. Full costs of HIV program with incorporation of a histoplasmosis screening and treatment program may be appropriate for a budgetary impact analysis. For a cost-effectiveness analysis, one would include the costs of the specific program of interest and the incremental costs and benefits are what are ultimately measured to assess cost-effectiveness.*

Warmly,

Radha Rajasingham, MD

Assistant Professor

Infectious Diseases & International Medicine

Department of Medicine, University of Minnesota
