# Supplementary material for: Cost-effectiveness evaluation of routine histoplasmosis screening among people living with advanced HIV disease in Latin America and the Caribbean
Source: PLOS Glob Public Health. 2023 Aug 15;3(8):e0001861. doi: 10.1371/journal.pgph.0001861 (PMC10427011; doi:10.1371/journal.pgph.0001861)
Supplement: Attachment — Submitted filename: LetterOfResponse5May2023.docx [file pgph.0001861.s013.docx]

May 5, 2023

Dear Dr. Kohler,

Thank you for your review of our manuscript entitled “Cost-effectiveness evaluation of routine histoplasmosis screening among people living with advanced HIV disease in Latin America and the Caribbean” PGPH-D-23-00160R1. See below for our point-by-point response.

Main comment

R2/sensitivity analyses: I concur with the reviewer and remain interested in the results of further sensitivity analyses on the modelling assumptions. Additional information on the dependency of the presented findings with respect to the chosen model parameters could strengthen the study. Even with a lack of data on the plausible variation of parameters, I would find a comprehensive sensitivity analysis informative, as it helps assess the robustness and determinants of the presented findings. I would like to encourage you to perform and include a comprehensive (one-way) sensitivity analysis as supplementary material and incorporate the main results of this analysis in the main text.

*Response: Thank you for this suggestion. We have performed additional sensitivity analyses and added the results to the supplementary material. Specifically we have evaluated the cost of Histoplasma antigen testing (at $20, $60, and $100 per test), Histoplasma antigen prevalence (ranging from 5% to 15%), and 30-day mortality among asymptomatic persons with histoplasmosis at 5%. These types of sensitivity analyses help the reader to understand a general trend. For example, in our model, if Histoplasma antigen testing cost $100 per test, then the ICER would be $280/life year saved (still highly cost-effective). Prevalence of Histoplasma antigen is inversely correlated with ICER, as is mortality. Hopefully these additional analyses help the readers to conceptualize the value/benefit of Histoplasma antigen screening.*

Minor comments

1. R1/Line 138: Please add the year/period in addition to the referenced study.

*Response: Thank you, the year 2020 has been added to this sentence.*

2. R2/HIV care: Consider incorporating the response in the manuscript as background information.

*Response: We are unclear to what this brief comment is referring. If the editor is able to clarify we would be happy to consider this further.*

3. R2/population finding: Consider presenting your chosen evaluation perspective, motivation for it, and implications of the evaluation perspective in the methods.

*Response: Thank you. Line 131 already stated:*

*The cost-effectiveness analysis was conducted from the perspective of the healthcare payer system, with costs and health outcomes discounted at 3% per year. Out of pocket costs incurred by patients such as transport, or loss of salary were not included.*

*We have added:*

*The perspective of the healthcare payer system was chosen as this would be most relevant to stakeholders considering investment in a histoplasmosis screening program.*

4. Consider aligning the formulation for the headings "Treatment for asymptomatic histoplasmosis-positive people" and "Management of symptomatic histoplasmosis" to emphasize that these sections cover the complementary aspects.

*Response: Thank you, these are better aligned now. Specifically, the first statement has been changed to:*

*Management of asymptomatic histoplasmosis*

5. It seems that only some, but not all revisions were tracked in version with track changes. Please resubmit a clean version and a version in which all text changes are tracked. Consider accepting formatting changes to create a version with tracked changes.

*Response: We have created a new version with tracked changes related to the above comments*. *We attempted to track changes to all changes in the prior version, but may have inadvertently not tracked everything. We apologize for any confusion.*

Warmly,

Radha Rajasingham, MD

Assistant Professor

Infectious Diseases & International Medicine

Department of Medicine, University of Minnesota
